# A home-school-doctor model to break the barriers for uptake of human papillomavirus vaccine

**DOI:** 10.1186/s12889-015-2269-1

**Published:** 2015-09-21

**Authors:** Albert Lee, Martin CS Wong, Tracy TN Chan, Paul KS Chan

**Affiliations:** JC School of Public Health and Primary Care, The Chinese University of Hong Kong, 4th Floor, School of Public Heath, Prince of Wales Hospital, Shatin, New Territories Hong Kong; Department of Microbiology, The Chinese University of Hong Kong, 1st Floor, Clinical Sciences Building, Prince of Wales Hospital, Shatin, New Territories Hong Kong; Centre for Health Education and Health Promotion, JC School of Public Health and Primary Care, The Chinese University of Hong Kong, 4th Floor, Lek Yuen Health Centre, Shatin, New Territories Hong Kong

**Keywords:** Human Papillomavirus vaccination, Adolescent girls, Vaccine uptake, Home-school-doctor model

## Abstract

**Background:**

A high coverage of human papillomavirus (HPV) vaccination is required to achieve a clinically significant reduction in disease burden. Countries implementing free-of-charge national vaccination program for adolescent girls are still challenged by the sub-optimal uptake rate. Voluntary on-site school-based mass vaccination programs have demonstrated high coverage. Here, we tested whether this could be an option for countries without a government-supported vaccination program as in Hong Kong.

**Method:**

A Home-School-Doctor model was evolved based on extensive literature review of various health promotion models together with studies on HPV vaccination among adolescent girls. The outcome measure was uptake of vaccination. Factors associated with the outcome were measured by validated surveys in which 4,631 students from 24 school territory wide participated. Chi-square test was used to analyze association between the categorical variables and the outcome. Multivariate analysis was performed to identify independent variables associated with the outcome with vaccine group as case and non-vaccine group as control.

**Results:**

In multivariate analysis, parental perception of usefulness of the Home-School-Doctor model had a very high odds ratio for uptake of HPV vaccination (OR 26.6, 95 % CI 16.4, 41.9). Paying a reasonable price was another independent factor associated with increased uptake (OR 1.71, 95 % CI 1.39, 2.1 for those with parents willing to pay US$125-250 for vaccination). For parents and adolescents who were not sure where to get vaccination, this model was significantly associated with improved uptake rate (OR 1.66, 95 % CI 1.23, 2.23). Concerns with side effects of vaccine (OR 0.70, 95 % CI 0.55, 0.88), allowing daughters to make their own decisions (OR 0.49, 95 % CI 0.38, 0.64) and not caring much about daughters’ social life (95 % CI 0.45, 0.92) were factors associated with a lower uptake.

**Discussion:**

The findings of this study have added knowledge on how a school-based vaccination program would improve vaccine uptake rate even when the users need to pay. Our findings are consistent with other study that the most acceptable way to achieve high uptake of HPV vaccine is to offer voluntary school-based vaccination.

**Conclusion:**

A model of care incorporating the efforts and expertise of academics and health professionals working closely with school can be applied to improve the uptake of vaccine among adolescent girls. Subsidized voluntary school-based vaccination scheme can be an option.

## Background

Many large scale studies have demonstrated the efficacy of human papillomavirus (HPV) vaccines [1, 2]. Achieving a clinically significant reduction in the incidence of cervical cancer requires a high coverage of vaccination [3–5]. The uptake rates still vary among countries with national HPV vaccination programs for adolescents, ranging from very high in UK and Australia of around 70 % [6, 7],medium (39 %) in Florida [8], and very low (17 %) in France [9]. ‘Strict’ school-linked mandate would be an option to improve uptake and a high coverage had also been achieved by voluntary on-site school-based mass programs [10] with one study demonstrating with 70 % coverage by year 8 and maximum vaccination coverage, 90 % by year 43 [11]. Parents were 2.5 to 4 times more likely to utilize school-based programs for immunization if their children received the last immunization at school than those children received vaccination at another site [12]. However school-based programs are not without barriers and oppositions.

Although the initial uptake rate of HPV vaccination program in school was 70 to 80 % in Australia for the first dose, the completion rates ranged from 55 to 77 % for the third dose [6]. In USA, the uptake rate among girls aged 13–17 was 25 % for the first dose, but only 6.9 % completed the third dose [13]. Publicly funded school-based program is not panacea for high uptake as parents still possess concerns over the vaccine [14]. Gamble et al. has summarized literatures reporting factors influencing familial decision regarding HPV vaccination and concluded that attitudes and recommendations of health care providers, and belief and attitudes of parents and adolescents were the key factors [15]. Other influential factors included interfacing with the health care system, peer norms, institutional policies and interventions such as mandate for school vaccination, and communication between parents and adolescents on sexual issues [15]. Health beliefs, developmental maturity, self-efficacy and relatedness to caregivers were found to be individual adolescent factors for uptake of the first dose,and response to the first dose, experience with health care system during the first visit, cost incurred obtaining the first dose, and evolving community belief about the vaccine and HPV were factors associated with adherence with the completion of the vaccine series according to conceptual framework by Katz et al. [16]. Bandura highlighted self-efficacy playing a role in the adoption of health behaviours in changing detrimental habits and maintaining the change [17]. Studies on cost-effectiveness of HPV vaccination should include willingness to pay to reflect whether the perceived values of benefits of consumers would exceed the retail prices of HPV vaccines [18].

Half of the cervical cancer cases globally are in Asia and but there are not many large-scale vaccination programs even among the well-developed parts of Asia, such as Taiwan, Hong Kong, Japan and South Korea [19]. Although those countries had high uptake rate for conventional vaccine-preventable diseases such as poliovirus or measles, uptake of HPV vaccine is very low [20]. In Hong Kong, studies by Choi et al. in 2008 and 2012 revealed that 2.4 % schools girls reported having received HPV vaccine and mothers reported 9.1 % their daughters been vaccinated respectively [21]. Only 7 % girls reported having received HPV vaccine by Lee et al. study in 2008 [22]. Lack of knowledge and information, concerns with efficiency and safety of vaccine, and not perceiving the risks of HPV infections were found to be commons reasons for not receiving the vaccine according to studies by Lee et al. and Li et al. in Hong Kong [22, 23]. These three recent Hong Kong studies have discussed the importance of school setting to equip adolescent girls with valid information concerning HPV vaccine as well as delivery of vaccine in school setting to facilitate the uptake rate [21–23].

Studies in Netherland and USA reported that receiving information from physicians and other community influencers was positively associated with HPV vaccine uptake [24–26]. Similarly in Hong Kong, multivariate analysis identified three independent significant factors, perceived threat of cancer, school providing more information on cancer prevention, and comments from relatives and friends having received the vaccine, were associated with initiation of vaccination [22]. HPV vaccine is currently not covered under the Government vaccination program in Hong Kong at the time of this study. A Home-School-Doctor Model would facilitate the initiation of HPV vaccine among adolescent girls by tackling the socio-cultural barriers as well as the financial and health care barriers. The model was based on the theory of leading models of health promotion as well as results of recent local studies and systematic review of overseas studies, in particular those conducted after approval of HPV vaccine [21–23, 27–34]. Our aim of study was to investigate whether availability of this model in school setting is an independent factor for parental decision to have their daughters being vaccinated. The study also aimed to identify other factors to be considered by parents independently associated with vaccine uptake of their daughters. The findings could add value to refine school-based vaccination program and also have an important impact to improve and sustain the uptake rate of vaccine.

## Methods

### Ethical statement

The study was approved by the Survey and Behavioral Research Ethics Committee of the Chinese University of Hong Kong. All clinical investigations have been conducted according to the principles expressed in the Declaration of Helsinki. All participants (parents or legal guardians) were asked to sign written consent for participation for the survey and the program if they decided to join in.

### Theoretical framework and survey instrument

Research hypotheses were generated on improvement of HPV vaccine uptake based on literature review of school-based HPV vaccine programs and the interfacing with health care systems [10, 14, 15, 17, 27–31, 33–35], and the conceptual framework for behavioral change models including vaccine uptake behavior [17, 32]. Study by van Keulen et al. on both daughters and mothers reported the importance of socio-psychological variables to the explained variance of HPV vaccination so future strategies to improve uptake should address attitudes, beliefs, subjective norms and habit strength [34]. The followings summaries the research hypotheses:

1. Mothers with correct understanding of HPV infection and causative link to cervical cancer and perception of seriousness of HPV infection and cervical cancer are more likely to have their daughters vaccinated;
2. Readily available information covering wider perspective of cervical cancer prevention including side effects and efficacy of HPV vaccine would improve uptake rate;
3. More discussion with family doctors would have motivating effect on uptake of HPV vaccine for adolescent girls;
4. Parents are willing to pay for vaccine if the cost is reasonable as well as social norm for vaccination as mode of prevention

**Fig. 1:**
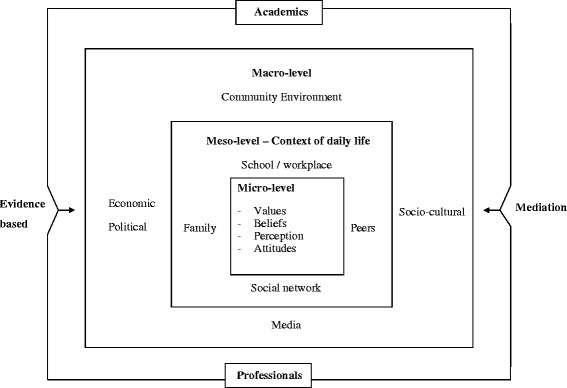
Framework for Health Behavioural Modification at different levels

Those research hypotheses led to evolution of the Home-School-Doctor model (Fig. 1) which is an ecological model of modification of health behaviours by acting at different levels from macro-level to meso-level and down to micro-level. At macro-level, the inputs from academics and professionals with evidence based practice would cultivate the socio-political environment and culture for HPV vaccination as preventive strategy for cervical cancer. This would also have impact on media and community awareness. This model also addresses the economic environment through subsidized scheme for vaccination through school setting. The changes at macro-level would enhance the joint effort of academic and medical professionals working through school setting and their social network to cultivate a positive social normamong the adolescent girls and their family members towards HPV vaccination as preventive strategy at meso-level in the context of their daily life. This would have impact at micro-level on individual beliefs, attitudes, values and perception leading to behavioural changes.

### Study design and data source

**Fig. 2:**
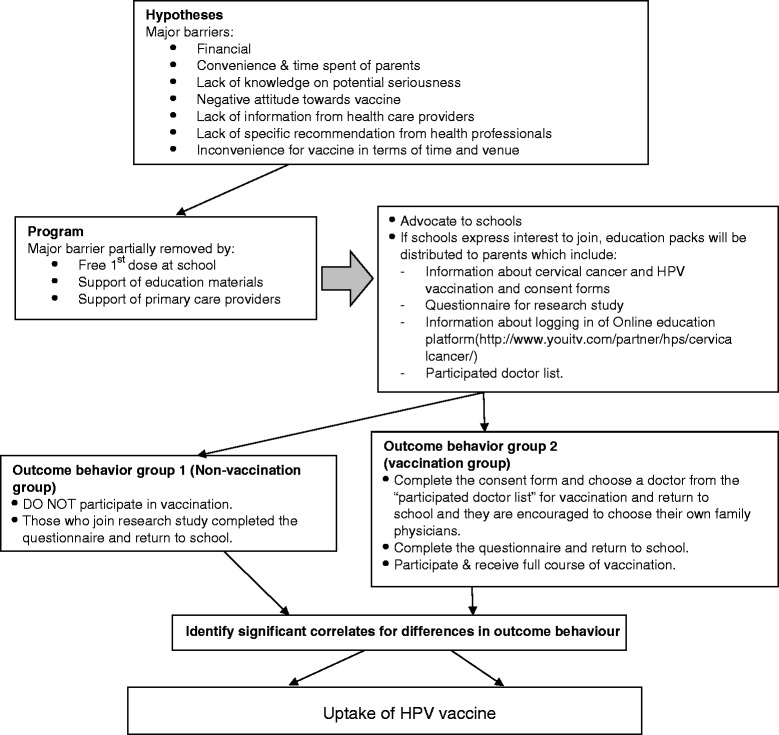
Testing of Intervention

Figure 2 summarizes the flow of the study, the program in addressing the research hypotheses and process of data collection for the outcomes. Purposeful sampling was adopted. Schools were invited to attend a health forum on cervical cancer prevention in July 2011. Those schools expressing interest to join the program during a school health forum were contacted, and a total of 24 schools from different districts of Hong Kong agreed to participate. The field study was carried out between November 2011 and May 2012. Each student received an education pack with concise information on prevention of cervical cancer, instruction to log on to an online education platform (http://www.youitv.com/partner/hps/cervicalcancer/), questionnaire, and information of vaccination program with an informed consent form. Parents and students were free to make their decisions on receiving vaccine.

Parents were invited to fill in a self-administered questionnaire as measuring tool. The survey tool was designed to measure the possible variables correlated with uptake of HPV vaccine based on the research hypotheses. The questionnaire also included a question as a variable by asking the parents whether the availability of the Home-School-Doctor model had a strong impact on their decisions to have their daughters vaccinated. The contents were refined by experts in public health and infectious diseases then pilot tested for face and content validation.

The model was introduced to all participating schools. Among all the students within the participating schools, those students receiving vaccination were classified as cases and those not having vaccination were control. The outcome was measured by uptake of vaccination or not. Odds ratios of the different variables were calculated by univariate and multi-variate analysis to distinguish the case and the control.

### Sample size

Based on the study in 2009 [22], the uptake rate of vaccination amongst female students was 7 %, the number of subjects needed to determine the proportion of uptake rate with precision set at 0.01 will be 2,604.

$$ \mathrm{N}\sim 1.9{6}^2 \times \mathrm{p}\left(1\hbox{-} \mathrm{p}\right)/\mathrm{precisio}{\mathrm{n}}^2 $$

In order to determine the number of subjects needed in each group analyzing the difference between the vaccinated group and non-vaccinated group, the sample size for each group will be:

$$ \mathrm{N}\sim 8 \times {\mathrm{P}}_0\left(1\hbox{-} {\mathrm{P}}_0\right)/\mathrm{precisio}{\mathrm{n}}^2 $$

If among the vaccinated group, 30 % expressed one particular predicting variable for vaccination (e.g., convenience) as important factor versus only 10 % among the non-vaccinated group, the P_0_ would then be 0.2. If the estimated precision is 0.02, the sample size of 3,200 is needed.

### Statistical analysis

Questionnaires were collected and descriptive data from all variables in the questionnaire were computed as either categorical or continuous variables. The proportion of students in each vaccination outcome behaviour group was tabulated. Chi-square test was used to analyze association between the categorical variables and the vaccination outcome behaviors. For those continuous variables, they were computed as scores. Cases were those subjects who had received the vaccination (outcome behaviour group 1). Non-vaccinated subjects were defined as control group (outcome behaviour group 2). Multivariate analysis was performed to identify independent variables associated with the cases, outcome behavior group 2. All data analysis was performed using SPSS version 14.0. P values of less than 0.05 were considered statistically significant.

## Results

**Fig. 3:**
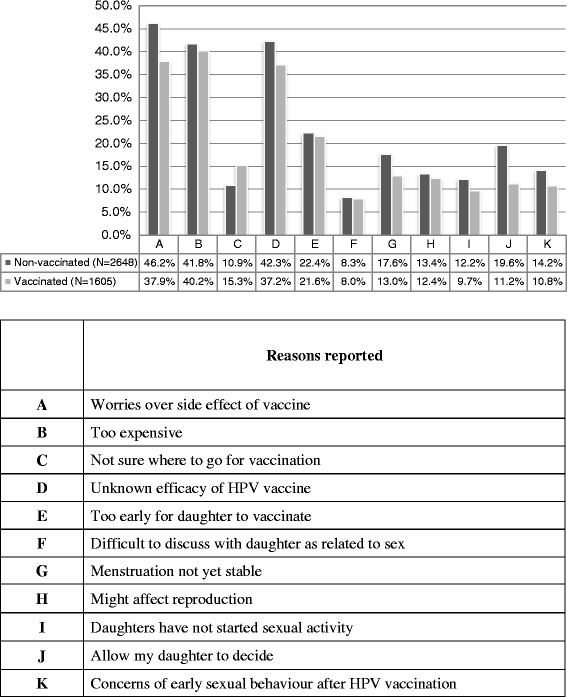
Reasons not refusing HPV vaccination in the past

A total of 4,631 students (response rate 61.4 %) returned from 24 schools situated in different districts of Hong Kong participated in the survey and from valid questionnaires 1,605 in vaccination group and 2,648 in non-vaccination group (Fig. 3). The characteristics of the study population with reference to the whole Hong Kong population are shown in Tables 1 and 2.

**Table 1:** Characteristics of the study participantscompared to the whole Hong Kong Population

Variables	Respondents (N)	Hong Kong Population
Total numbers of schools	24	1018
Number of primary schools	12	528^a^
Number of secondary schools	12	490^a^
Total numbers of students	4631	736,229
Number of primary schoolsstudents	687	317,442^a^
Number of secondary schools students	3944	418,787^a^
Level of education of respondents		
Primary school level or below^c^	11.0 %	29.4 %^b^
Secondary school level^c^	72.6 %	54.8 %^b^
Tertiary or above	16.4 %	15.8 %^b^

^a^Education and Manpower Bureau, HKSAR (Hong Kong)http://www.edb.gov.hk/tc/about-edb/publications-stat/figures/index.html (Last revision date: 09 July 2013)

^b^Census and Statistics Department, HKSAR (Hong Kong), 2011

^c^Primary education 6 years, secondary education 7 years

**Table 2:** Age of respondents and schools distribution

Age of respondents	In primary schools	In secondary schools
Age 30 or below	16 (2.4 %)	192 (5.0 %)
Age 31–40	267 (40.0 %)	890 (23.0 %)
Age 41 or above	385 (57.6 %)	2785 (72.0 %)
Primary & secondary schools distribution^b^ (by district)	Schools in HK	Schools joined HPV program
HK South	87 (7 %)^a^	2 (8 %)
HK East	61 (5 %)^a^	2 (8 %)
Kowloon City	107 (8 %)^a^	3 (13 %)
Wong Tai Sin	68 (5 %)^a^	1 (4 %)
Kwun Tong	83 (6 %)^a^	2 (8 %)
TuenMun	89 (7 %)^a^	4 (17 %)
Yuen Long	112 (9 %)^a^	2 (8 %)
Tai Po	54 (4 %)^a^	1 (4 %)
Sai Kung	68 (5 %)^a^	3 (13 %)
Kwai Tsing	108 (8 %)^a^	4 (17 %)

^a^School Lists (By District) (2013/14) (Last Updated: Oct 2013), Education and Manpower Bureau, HKSAR (Hong Kong) http://applications.edb.gov.hk/schoolsearch/schoolsearch.aspx?langno=1

^b^Primary education 6 years, secondary education 7 years

Figure 3 shows the reasons why the parents refused HPV vaccination for their daughters as well as reasons for not sending them for vaccination. Uni-variate analysis revealed that the impact of Home-School-Doctor model, willingness to pay, self-perceived knowledge on HPV vaccination, understanding the link between HPV and cervical cancer, information from and discussion with doctors, information from friends/relatives or media, and not sure where to get vaccination were factors associated with a higher uptake of HPV vaccine (Table 3). Conversely, uncertainty of effectiveness, concerns of early sexual behaviors after HPV vaccination, worries over side-effects, concerns of effect on reproduction and menstruation, daughters not yet started sexual activity, misconception of cervical cancer, letting daughters to make their own decisions, and never heard of HPV were factors associated with a lower uptake of HPV vaccine. In multi-variate analysis, the impact of Home-School-Doctor model was found to be the strongest association for uptake of HPV vaccination (OR 26.6.6, 95 % CI16.44, 41.96) (Table 4). Paying a reasonable price was also another independent factor associated with uptake rate, with parents willing to pay between HK$1,000 to 2,000 (US$125 to 250) having 1.71odds (95 % CI 1.39, 2.1) for uptake of vaccine (Table 4). If parents and adolescents were not sure where to get vaccination, the availability of this model became an important factor for them to take up the vaccine (OR 1.66, 95 % CI 1.23, 2.23) (Table 4). Concerns with side effects of the vaccine, allowing daughters to make their own decisions and not caring much on daughters’ social life were all significant independent factors associated with a lower uptake of the vaccine (Table 4).

**Table 3:** The association between parents’ response to questionnaire and uptake of HPV vaccine by school girls using univariate analysis

	OR	95 % CI	P
Availability of the current Home-School-Doctor model			
No	1	Reference	
Yes	36.09	(24.68, 52.79)	<0.001
Amount (HK$) willing to pay for a full course			
<1,000	1	Reference	
1,001–2,000	2.02	(1.75, 2.33)	<0.001
>2,001	1.70	(1.42, 2.03)	<0.001
Parents self-perceived knowledge on HPV vaccine			
Very insufficient, insufficient & somewhat insufficient	1	Reference	
Somewhat sufficient	1.40	(1.21, 1.63)	<0.001
Sufficient & very sufficient	1.59	(1.35, 1.88)	<0.001
Not sure where to go for vaccination			
No	1	Reference	
Some importance as a factor to consider	1.30	(1.11, 1.53)	0.002
Important/very important	1.44	(1.19, 1.74)	<0.001
I have frequent chat with daughter			
No	1	Reference	
Yes	1.34	(1.11, 1.61)	0.002
Consider protection sustainability in choosing HPV vaccine			
No	1	Reference	
Yes	1.31	(1.16, 1.49)	<0.001
Consider protection to cervical cancer inchoosing HPV vaccine			
No	1	Reference	
Yes	1.26	(1.11, 1.44)	<0.001
Vaccine can produce antibodies against HPV			
No	1	Reference	
Yes	1.21	(1.07, 1.37)	0.002
Cervical cancer is caused by HPV			
No	1	Reference	0.001
Yes	1.26	(1.10, 1.45)	
I would discuss about prevention of cervical cancer with doctor			
Definitelyb not/No/Maybe not	1	Reference	
Yes/Maybe	1.11	(0.96, 1.28)	0.16
Yes/Definitely yes	1.21	(1.03, 1.42)	0.02
Heard about HPV vaccine from TV/magazine			
No	1	Reference	
Yes	1.44	(1.21, 1.71)	<0.001
Heard about HPV vaccine from school			
No	1	Reference	
Yes	1.20	(1.02, 1.42)	0.03
Never heard about HPV vaccine			
No	1	Reference	
Yes	0.55	(0.36, 0.83)	0.005
Worries over side effect of vaccine			
No	1	Reference	
Some importance as a factor to consider	1.07	(0.90, 1.29)	0.45
Important/Very important	0.62	(0.52, 0.74)	<0.001
Menstruation not yet stable			
No	1	Reference	
Some importance as a factor to consider	0.93	(0.80, 1.08)	0.35
Important/Very important	0.62	(0.51, 0.74)	<0.001
Allow my daughter to decide			
No	1	Reference	
Some importance as a factor to consider	0.57	(0.49, 0.68)	<0.001
Important/Very important	0.40	(0.33, 0.48)	<0.001
Unknown efficacy of HPV vaccine			
No	1	Reference	
Some importance as a factor to consider	1.10	(0.92, 1.31)	0.29
Important/Very important	0.73	(0.62, 0.86)	<0.001
Too early for daughter to vaccinate			
No	1	Reference	
Some importance as a factor to consider	0.82	(0.70, 0.96)	0.012
Important/Very important	0.80	(0.68, 0.94)	0.006
Difficult to discuss with daughter as related to sex			
No	1	Reference	
Some importance as a factor to consider	0.81	(0.68, 0.98)	0.027
Important/Very important	0.85	(0.68, 1.07)	0.16
Might affect reproduction			
No	1	Reference	
Some importance as a factor to consider	0.74	(0.62, 0.87)	<0.001
Important/Very important	0.77	(0.64, 0.93)	0.008
Daughters have not started sexual activity			
No	1	Reference	
Some importance as a factor to consider	0.64	(0.53, 0.78)	<0.001
Important/Very important	0.65	(0.53, 0.80)	<0.001
Concerns of early sexual behaviour after HPV vaccination			
No	1	Reference	
Some importance as a factor to consider	0.73	(0.61, 0.88)	0.001
Important/Very important	0.62	(0.51, 0.76)	<0.001
I do not care about daughter’s social life			
No	1	Reference	
Yes	0.66	(0.51, 0.86)	0.0021
Cervical cancer is inherited			
No	1	Reference	
Yes	0.72	(0.60, 0.86)	<0.001
Cervical cancer happens only on those with multiple sex partners			
No	1	Reference	
Yes	0.80	(0.66, 0.98)	0.033

**Table 4:** Association between parents’ response to questionnaire and uptake of HPV vaccine by school girls using multi-variate analysis

	OR	95 % CI	P
Availability of Current Home-School-Doctor Model			
No	1	Reference	
Yes	26.6	(16.44, 41.96)	<0.001
Amount (HK$) willing to pay for a full course			
<1,000	1	Reference	
1,001-2,000	1.71	(1.39, 2.10)	<0.001
>2,000	1.39	(1.05, 1.84)	0.023
Not sure where to go for vaccination			
No	1	Reference	
Some importance as a factor to consider	1.45	(1.14, 1.84)	0.003
Important/ Very important	1.66	(1.23, 2.23)	0.001
Allow my daughter to decide			
No	1	Reference	
Some importance as a factor to consider	0.59	(0.46, 0.74)	<0.001
Important and very important	0.45	(0.34, 0.58)	<0.001
Do not care about daughter’s social life			
No	1	Reference	
Yes	0.62	(0.41, 0.94)	0.026
Consider protection sustainability in choosing HPV vaccine			
No	1	Reference	
Yes	1.21	(1.00, 1.46)	0.048
Parents self-perceived knowledge on HPV vaccine			
Very insufficient, insufficient & somewhat insufficient	1	Reference	
Somewhat sufficient	1.20	(0.96, 1.50)	0.11
Sufficient & very sufficient	1.38	(1.05, 1.80)	0.020

## Discussion

The findings of this study have added knowledge on how a school-based vaccination program would improve vaccine uptake rate even when the users need to pay. The study has identified independent factors associated with higher or lower HPV vaccine uptake based on multi-variate analysis. The ‘Home-School-Doctor’ model had very strong impact on parents’ decision for their daughter to opt for HPV vaccine with a very high odds ratio (OR 26.6). Families willing to pay HK$1,000 (US$125) or more for a full course of vaccination were also more likely to take up the vaccine. The availability of the model increased parents’ likelihood to consider vaccination if they were uncertain of possible venues for vaccination. Worries over side effects were significant independent factors associated with low uptake as well as those parents allowing their daughters to make decision and not caring too much about their social life.

A systematic review published in 2012 identified factor such as coverage by health insurance, more health care utilization particularly for preventive care, having health care providers as source of information, positive attitude towards the vaccine and higher vaccine related knowledge associated with HPV vaccine uptake among teenage girls [24]. The systematic review also noted considerably higher uptake rate in settings with a school-based program. [6, 14, 17, 27, 36–38]. Our findings are consistent with study by Skinner and Copper Robins that the most acceptable way to achieve high uptake of HPV vaccine is to offer voluntary school-based vaccination [10]. Skinner and Cooper Robins also addressed voluntary on site-school-based mass vaccination as one of the evidence-based approaches to increase vaccine uptake among adolescents apart from strict mandate for vaccination [10]. Strict mandates have been debated with controversies in USA as genital wart is exclusively sexual transmitted so infected individuals do not pose an immediate risk to others in school setting [39, 40].

The systematic review also highlighted the important role of primary care providers in providing HPV-related information and assisting girls to initiate and/or complete the vaccine series. If both school setting and primary care setting become reliable source of information for parents and teenage girls, this would have significant impact on the beliefs, attitudes and intention regarding HPV vaccination. Hong Kong study in 2009 reported that school providing information on cervical cancer prevention had significantly motivated their consideration for HPV vaccination [22]. Study among Japanese mothers highlighted the importance of physicians actively addressing the safety concerns and the necessity for the vaccine at particular age to achieve high uptake even in publically funded HPV vaccine program [41]. Our Home-School-Doctor model introduced the vaccine to parents via school setting through academic institutions in partnership with physicians in the local catchment area. The support of medical professional organization in mobilizing district network of doctors not only conveyed a strong message of endorsement to the public, but also added confidence to both primary care providers and the recipients (parents and teenage girls). Studies have shown a consistent finding that primary care providers are less comfortable in vaccinating younger versus older adolescents, and thus the endorsement of vaccination by a professional organization is of great importance [42]. The Home-School-Doctor model has an additional endorsement from academic institution which could be important in the context where Government’s official support is still lacking.

Paper by Tissot et al. addressed the needs of educating paediatricians about the high prevalence of HPV among all sexually active adolescents and universal recommendation for the vaccine [42]. That study revealed the need to enhance access to vaccination by providing HPV vaccines in alternative settings, addressing specific educational needs related to HPV and HPV vaccines, and providing pediatricians with guidance as to how to address the specific concerns that parents might have about a vaccine for sexually transmitted disease. School can therefore be an alternative setting. It is often difficult to identify the educational needs for both consumers and health care providers as they are from diverse background, especially the consumers. One would try to have fact sheets with credible information provided by academic institution delivered via school setting matching the educational needs of students and parents. Studies have also shown the effectiveness of school-based health promotion programs in changing health behaviors including sexual behaviors [43], and improvement of health literacy [44]. The online education video incorporated in our model not only allowed parents and students to explore at their time of convenience, it also enhanced their confidence with messages from doctors in tackling the common myths regarding the vaccine and prevention of cervical cancer. Both printed and online materials could act as supplementary information for schools in developing comprehensive school health education on reproductive health and disease prevention. A study by Garland et al. reported the high coverage rate of HPV vaccine in Australia after commencing the school-based government vaccination program since 2007 [45]. The study also highlighted the challenge around the public perceptions of the appropriateness and safety of vaccinating large numbers of children in school setting. The traditional separation of the roles of health and education sectors regarding immunization poses further challenges as students might not be well informed, or the relevant information might not be readily accessible to them. Parents and students might not have the opportunity to ask further questions. This Home-School-Doctor model has tried to resolve some of these challenges by professional inputs and endorsement from doctors and also academic institutions. It would help those parents initially uncertain where to go for vaccination as reflected by higher vaccine uptake of their daughters (Table 3).

Parents expressing their wishes to let daughters to make their own decisions or not caring much about the social life of their daughters were found to be less likely to join the program. Women with no sex experience usually reported not being at risk as the main reason for not wanting the vaccination [46]. The prevalence of early sexual experience among adolescent girls in Hong Kong is not high [47]. If parents left the decision entirely to their daughters, they would perceive themselves not being at risk and not taking up the vaccination during early adolescence for better protection.

This model has also provided educational opportunities for health care providers. Seminars were organized for them with the latest update on HPV vaccine covering safety, duration of immunity, contraindications and description of HPV-related diseases. The seminars also provided practical tips in raising sensitive issues with teenage girls. A recent study from Hong Kong has revealed a low level of knowledge on HPV infection among primary care physicians, and their prescription of HPV vaccine was hindered by the perceived parental concerns, and they relied passively on Government recommendations [48]. The opportunity for doctors to give health talk to schools in this program would help the doctors to gain better understanding on the needs and concerns of parents and young girls, thus further providing reassurance to them. Information is also available in CD format for those doctors who could not attend the seminars. Fulfilling the education needs of primary care providers is important as this will indirectly improve communication to the consumers. Health professionals remain central in influencing parents’ decision and parental intention to have their daughters vaccinated approximates the uptake rate [49].

Cost has been identified as an important factor in many studies [35], therefore the subsidized price would help to boost up the vaccination uptake among parents. US study by Brown et al. revealed mean willingness to pay was US$560 on average for 80 % cervical cancer prevention for duration of 10 years even without added protection against genital warts [178. The GDP per capita in Hong Kong is comparable to USA (US$36,667 and US$49,922, respectively, according to International Monetary Union, 2012) so one would target those families willing to pay US$250 for the full course (Table 4). The current market price of three doses of HPV vaccine in Hong Kong is around US$400. Whilst the Hong Kong Government does not provide a free-of-charge HPV vaccination program, it could consider a subsidized scheme in school setting similar to our model.

Apart from cost, school-based vaccination is a complex initiative with many logistic factors needs to be considered. These include dissemination, collection and recording of consent forms, management of adolescent anxiety on site, as well as education of teachers, parents and students about vaccination [45]. Our model managed to address some of those complex logistic factors smoothly, which was the key to success. While the setting of schools and colleges would offer special opportunities for vaccine delivery, school attendance is variable particularly in less well developed countries [50]. This current model would lead us to consider adopting the concept of healthy setting approach to promote health in the context of their living environment as school is only one example of setting. One would consider non-traditional, non-institutional settings such as social venues for youth relevant to particular culture to ensure that the settings approach would respond to societal changes and addresses inequalities [51].

There is strong evidence that engagement of adolescents would lead to higher compliance with medical advice [45]. Adolescents who had the energy and willpower to take care of themselves, complied with health regimens with a 6.69 fold likelihood compared to adolescents who did not have energy and willpower [45]. Our study showed that those parents wanting to leave decision to their daughter or not wanting to deal with their social life were less likely to opt for HPV vaccination for their daughters. More active engagement and direct involvement with adolescents is another strategy for improvement of HPV vaccination among the young age group. In fact, many of the participating schools continued to arrange HPV vaccination using this model.

There are several limitations to this study. The participation of schools, students and parents was voluntary, so there could be a volunteer bias. The self-selection rather than random selection could affect the representativeness of our study population. Nevertheless, if a lot of randomly selected schools declined and not co-operated with the protocol, this would forfeit the aims of the study. Our study population consisted of schools from different parts of the territory with higher proportion from districts which were not in metropolitan areas, and a substantial proportion came from lower socio-economic class. We also noticed that the education level of parents in this study was higher comparable to whole Hong Kong population.

We only used the completion of second dose at primary care clinic as a measurement of uptake rate. As the families needed to pay for second dose and they chose their own primary care clinics, they were highly unlikely to default the third dose. The research team used SMS message to remind them for the second and third dose, and their primary providers also had reminder mechanism. Although they all received the first dose free and needed to pay for the second dose, and yet over 80 % of the students attended for second dose at their chosen primary care clinics. Once the primary care providers have established rapport with their patients, their compliance to preventive health services in primary care setting is very high [51, 52, 53].

## Conclusion

A model of care incorporating the efforts and expertise of academics and health professionals working closely with school setting can have great impact on uptake of HPV vaccine among adolescent girls, making other barriers relatively insignificant. This model could address the unmet educational and health needs of parents, students and school educators. Some of those participating schools had self-initiated similar vaccination program the year after, and there are still on-going enquiries from those schools as well as schools not participated previously. Voluntary school-based vaccination at subsidized rate would be a model to promulgate and sustain the HPV vaccination program if a free national program is not feasible. Continuous research is needed to refine the delivery model in meeting the complex needs of all stakeholders.

### What is ready known to this topic?

The uptake of HPV vaccine among adolescents varies among countries with universal coverage. Inadequate knowledge of cervical cancer prevention, concerns of vaccine efficacy and side effects, lack of recommendations from health professionals have been found as major barriers. The voluntary school based vaccination programs have shown to improve uptake rate.

### What this study adds?

This is the first study conducted in a country without national vaccination program to test the efficacy of a non-Government-led school-based program. The Home-School-Doctor model addressed the main barriers including the financial concerns. It was found that adoption of the model is substantially more likely to be associated with HPV vaccine uptake, and this bears strong implications for healthcare professionals and school stakeholders to design and implement this model in school settings via an organized, concerted effort to improve vaccine uptake rates.
